# Achilles Tendon Morphology Is Related to Triceps Surae Muscle Size and Peak Plantarflexion Torques During Walking in Young but Not Older Adults

**DOI:** 10.3389/fspor.2020.00088

**Published:** 2020-08-06

**Authors:** Katherine R. Knaus, Anahid Ebrahimi, Jack A. Martin, Isaac F. Loegering, Darryl G. Thelen, Silvia S. Blemker

**Affiliations:** ^1^Department of Biomedical Engineering, University of Virginia, Charlottesville, VA, United States; ^2^Department of Mechanical Engineering, University of Wisconsin-Madison, Madison, WI, United States; ^3^Department of Orthopedics and Rehabilitation, University of Wisconsin-Madison, Madison, WI, United States; ^4^Department of Biomedical Engineering, University of Wisconsin-Madison, Madison, WI, United States; ^5^Department of Mechanical and Aerospace Engineering, University of Virginia, Charlottesville, VA, United States

**Keywords:** aging gait, gastrocnemius, soleus, plantarflexion torque, muscle volume, tendon cross-sectional area

## Abstract

The interaction of the triceps surae muscles and the Achilles tendon is critical in producing the ankle plantarflexion torque required for human walking. Deficits in plantarflexor output are a hallmark of reduced mobility in older adults and are likely associated with changes in the triceps surae muscles that occur with age. Structural differences between young and older adults have been observed in the Achilles tendon and in the triceps surae muscles. However, less is known about how age-related differences in muscle and tendon morphology correspond with each other and, furthermore, how those morphology differences correlate with age-related deficits in function. The goal of this work was to investigate whether there is a correlation between age-related differences in triceps surae muscle size and Achilles tendon cross-sectional area (CSA) and whether either is predictive of ankle plantarflexion torque during walking. We used magnetic resonance imaging (MRI) to measure triceps surae muscle volumes and tendon CSAs in young (*n* = 14, age: 26 ± 4 years) and older (*n* = 7, age: 66 ± 5 years) adults, and we determined peak plantarflexion torques during treadmill walking. We found that individual muscle volumes as a percentage of the total triceps surae volume did not differ between young and older adults, though muscle volumes per body size (normalized by the product of height and mass) were smaller in older adults. Achilles tendon CSA was correlated with body size and muscle volumes in young adults but not in older adults. The ratio of tendon CSA to total triceps surae muscle volume was significantly greater in older adults. Peak ankle plantarflexion torque during walking correlated with body size and triceps surae volume in young and older adults but was correlated with tendon CSA only in the young adults. Structure–function relationships that seem to exist between the Achilles tendon and the triceps surae muscles in young adults are no longer evident in all older adults. Understanding mechanisms that determine altered Achilles tendon CSA in older adults may provide insight into age-related changes in function.

## Introduction

The interaction of the triceps surae muscles with the Achilles tendon is critically important to human walking. This complex muscle–tendon interplay is the primary source of plantarflexion torque at the ankle and is implicated in the development of walking deficits that occur with age. Gait differences between young and older adults are clearly associated with differences in plantarflexor output (Winter et al., 1990; Kerrigan et al., 1998; DeVita and Hortobagyi, 2000; Boyer et al., 2017). Numerous studies have investigated age-related differences in the morphology and the mechanical properties of the Achilles tendon and triceps surae muscles (Karamanidis and Arampatzis, 2006; Onambele et al., 2006; Stenroth et al., 2012), but it remains unclear how muscle and tendon structure relate to each other in both young and older adults. Further, the association between age-related changes in muscle and tendon structure and changes in plantarflexor output has not been fully explored.

The triceps surae muscles share a common series elastic element in the Achilles tendon but are an anatomically complicated group of muscles that may experience complex changes with age. The biarticular gastrocnemius and uniarticular soleus muscles within this group differ greatly in volume (Ward et al., 2009; Handsfield et al., 2014), architecture (Ward et al., 2009; Rana et al., 2013; Dalmau-Pastor et al., 2014; Bolsterlee et al., 2019), and fiber type (Johnson et al., 1973). It is not surprising that empirical and modeling studies suggest functional differences between these muscles, predicting unequal contributions to propulsion and support during walking (Neptune et al., 2001; Anderson and Pandy, 2003; McGowan et al., 2008; Francis et al., 2013). Each muscle's function is rooted in its intricate architecture. The gastrocnemius comprises distinct medial and lateral heads with different origins (Dalmau-Pastor et al., 2014). The soleus is divided by its aponeuroses into bipennate anterior compartments and unipennate posterior compartments (Agur et al., 2003; Hodgson et al., 2006; Bolsterlee et al., 2018). Age-related sarcopenia leads to reduced muscle volumes in the triceps surae as a whole (Janssen et al., 2000; Morse et al., 2005); however, it is unclear whether sarcopenia similarly affects each individual triceps surae muscle. It is possible that the individual muscles' susceptibility to atrophy may vary due to differences in mechanical stimuli and fiber-type composition (Nilwik et al., 2013).

Triceps surae muscle volume is indicative of ankle plantarflexion torque capacity (Fukunaga et al., 2001), such that a smaller triceps surae volume may explain why older adults employ lower ankle plantarflexion torques than young adults when walking at the same speed (DeVita and Hortobagyi, 2000; Franz and Thelen, 2015). However, direct comparisons between plantarflexion torques during walking and triceps surae muscle volumes have not been performed across age groups to assess age-related differences. Further, given the functional differences between triceps surae muscles, age-related changes in individual components of this muscle group may better explain torque deficits.

In contrast to changes in muscle size, Achilles tendon cross-sectional area (CSA) is maintained or even increased with age (Onambele et al., 2006; Stenroth et al., 2012). Healthy tendons adapt to changes in mechanical loading (Bohm et al., 2015), suggesting that the ratio of muscle size to tendon size should remain constant. However, the correlation between muscle size and tendon size has not been well studied, nor have age-related differences in this correlation been observed. Understanding the morphological relationship between the Achilles tendon and individual components of the triceps surae, in addition to the full muscle group, may provide further insight into age-related changes in walking.

The goal of this work was to investigate how triceps surae muscle volumes collectively and individually differed with age. We aimed to determine (1) if age-related differences in muscle size correlated with age-related differences in Achilles tendon CSA and (2) if age-related differences in muscle size and tendon CSA were predictive of age-related differences in joint torque during walking. We used magnetic resonance imaging (MRI) to measure muscle volumes and tendon CSAs in young and older adults and determined peak plantarflexion torques during treadmill walking. We tested the hypotheses that (i) triceps surae muscle volumes would negatively correlate with age and positively correlate with body size while Achilles tendon CSA would positively correlate with both age and body size, (ii) age-related changes in volume would differ between individual muscles of the triceps surae, and (iii) differences in tendon and muscle size would positively correlate with differences in ankle plantarflexion torque during walking in both young and older adults.

## Methods

### Subjects

Fourteen healthy young adults (six females/eight males, age: 26 ± 4 years, height: 1.78 ± 0.10 m, mass: 74.87 ± 12.11 kg) and seven healthy older adults (four females/three males, age: 66 ± 5 years, height: 1.76 ± 0.07 m, mass: 74.57 ± 15.26 kg) participated in this study (Table 1). No subjects had a history of orthopedic or neurological impairment or injury to the lower limb, and all could walk comfortably on a treadmill. All older adult subjects reported participating in daily physical activity. All subjects provided written consent, and the study protocol was approved by the University of Wisconsin–Madison Health Sciences Institutional Review Board.

**Table 1:** Subject characteristics (mean ± standard deviation).

	**Age (years)**	**Height (m)**	**Mass (kg)**	**Tendon CSA (mm^**2**^)**	**Moment arm (mm)**
Young	25.5 ± 4.3	1.78 ± 0.1	74.87 ± 12.11	61.35 ± 12.24	46.64 ± 3.87
Older	66 ± 4.8	1.76 ± 0.07	74.57 ± 15.26	65.54 ± 6.96	43.54 ± 8.13
*p*-value	**0.0003**	0.601	0.911	0.248	0.086
	**Peak ankle torque (Nm)**	**Stride length (m)**	**Step length (m)**	**Stride time (s)**	**Cadence (steps/min)**
Young	114.23 ± 24.01	1.38 ± 0.09	0.69 ± 0.04	1.11 ± 0.07	108.74 ± 6.83
Older	106.04 ± 27.72	1.27 ± 0.1	0.64 ± 0.05	1.02 ± 0.08	118.48 ± 9.84
*p*-value	0.478	**0.036**	**0.035**	**0.036**	**0.040**

*All gait characteristics are reported for subjects walking at a speed of 1.25 m/s. Young and older adults did not differ significantly in body size, Achilles tendon geometry, or peak torque produced during walking. However, young and older adults did differ in their gait spatiotemporal parameters, with older adults using shorter strides at a higher cadence than young adults walking at 1.25 m/s. p < 0.05 was considered significant and written in bold*.

### Triceps Surae Muscle Volume Measurements

The triceps surae muscles and Achilles tendon were imaged with a 3-T Signa PET/magnetic resonance (MR) scanner (GE Healthcare) using a spoiled gradient recall-echo sequence that used iterative decomposition of water and fat with echo asymmetry and least squares estimation (IDEAL-SPGR) (Reeder et al., 2007). Subjects lay supine with their right ankle relaxed and wrapped in a GEM Medium Flex Coil. Two sets of three-dimensional images were collected with the following scanning parameters: in-plane resolution: 0.72 × 0.72 mm; slice thickness: 2 mm; imaging matrix: 512 × 512 × 76; flip angle: 14°. Continuous axial images were obtained from the calcaneus to the femur proximal to the condyles.

**Figure 1:**
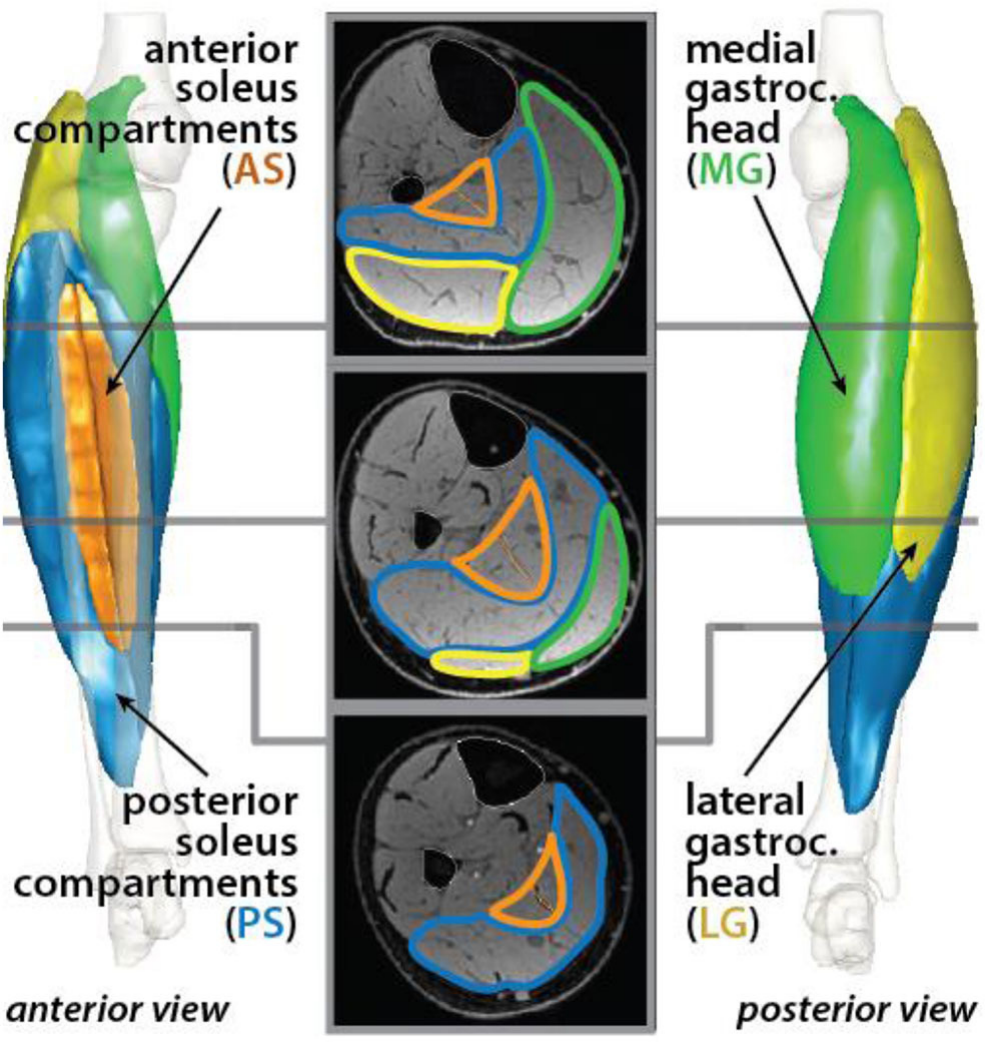
Triceps surae muscles were segmented in axial MR images. The muscle cross sections were defined for the medial (green) and lateral (yellow) heads of the gastrocnemius and the posterior (blue) and anterior (orange) compartments of the soleus. Each muscle was reconstructed in 3-D.

The triceps surae muscles in the right limb were segmented using a Matlab (MathWorks Inc., Natick, MA, USA) software package that was developed in the Multiscale Muscle Mechanophysiology lab at the University of Virginia for measuring lower-limb muscle volumes (Handsfield et al., 2014). In each axial image, a single researcher manually outlined the boundaries of four unique muscles: the medial gastrocnemius (MG), the lateral gastrocnemius (LG), the posterior soleus (PS), and the anterior soleus (AS) (Figure 1). The heads of the gastrocnemius and the soleus muscles were identified based on a detailed slice-by-slice segmentation atlas of lower-limb muscles (Handsfield et al., 2014), and the posterior and anterior compartments of the soleus were identified based on descriptions of the aponeurosis that visibly separates these regions of the muscle (Hodgson et al., 2006; Bolsterlee et al., 2018). Individual muscle volumes were calculated by summing the volume of voxels from the segmentation in each slice. Previously, we determined the average intra-user variability in measuring muscle volumes using this segmentation method to be 4.4% (Handsfield et al., 2016). Total triceps surae muscle volume was defined as the sum of the volumes of the four individual muscles. Relative muscle volumes were determined by dividing individual muscle volumes by the total triceps surae muscle volume.

To determine differences in muscle size independent of body size differences, we normalized individual and total muscle volumes by dividing by the product of subject height and mass. Triceps surae muscle volumes have been shown to vary with height * mass as a metric of body size in healthy adults (Handsfield et al., 2014).

### Achilles Tendon CSA Measurements

The Achilles free tendon was also segmented in the axial MR images from the most proximal image where the calcaneus was visible to the soleus muscle–tendon junction (MTJ), which was defined as the most distal image where the soleus was visible. The tendon volume was determined from the summed CSA multiplied by the slice thickness, while the tendon length was computed as the summed distance between the centroids of adjacent cross sections (Handsfield et al., 2014).

To test our hypotheses, a representative Achilles CSA was determined from the middle slice of the free tendon. A ratio of tendon size per muscle size was calculated by dividing Achilles tendon CSA by individual and total triceps surae muscle volumes, just as tendon size per body size was found by dividing tendon CSA by the product of height and mass.

### Achilles Tendon Moment Arm Measurements

Achilles tendon moment arms were measured using a previously described method combining ultrasonography and motion capture (Rasske et al., 2017; Keuler et al., 2019; Ebrahimi et al., 2020). Briefly, subjects lay prone with their right knees flexed 20° while their ankles were rotated from maximum dorsiflexion to maximum plantarflexion. Subjects were asked to provide resistance during ankle rotation to engage their triceps surae muscles. An ultrasound transducer positioned over the Achilles tendon was used to collect B-mode images. The superficial and deep edges of the tendon were manually identified, and a tendon line of action was determined from the best fit between them. Marker clusters on the shank, foot, and transducer were used to record kinematics in order to transform ultrasound images into the reference frame of the shank. A best-fit screw axis that described the foot motion with respect to the shank was computed to define a functional axis (Siston et al., 2005). Achilles tendon moment arm was computed in each image frame as the perpendicular distance between the tendon line of action and the functional axis (Wade et al., 2019). The moment arm was estimated for a 0° posture using a quadratic fit of moment arm relative to ankle angle.

### Peak Ankle Torque Measurements During Walking

Subjects walked on an instrumented treadmill (sample rate: 1,900 Hz, Bertec Corp.) at 1.25 m/s. Ground reaction forces were recorded during at least two 10-s trials (minimum 10 strides). Motion capture (sample rate: 190 Hz, Motion Analysis Corp.) was used to record 3-D trajectories of markers positioned on the pelvis, thigh, and shank during walking. Lower-extremity kinematics and kinetics were computed using standard inverse dynamics techniques (Visual3D, C-Motion, Inc.). Peak ankle plantarflexion moments were averaged across gait cycles for each subject. Plantarflexion torque was assumed to be generated entirely by the triceps surae muscles. Peak ankle torque was divided by Achilles tendon moment arm to provide an estimate of Achilles tendon force at these peaks.

### Statistical Analyses

We used a Mann–Whitney rank sum test, a non-parametric test, to determine differences in our measurements between young and older subject groups. For tests that were repeated over the four individual muscles, we corrected for family-wise error rate using the Holm–Bonferroni method. Linear regression analysis was used to determine correlations between different measurements in either young or older subjects. Significance was set at *p* = 0.05.

## Results

### Relative Volumes of Triceps Surae Muscles Did Not Differ Between Young and Older Adults

**Figure 2:**
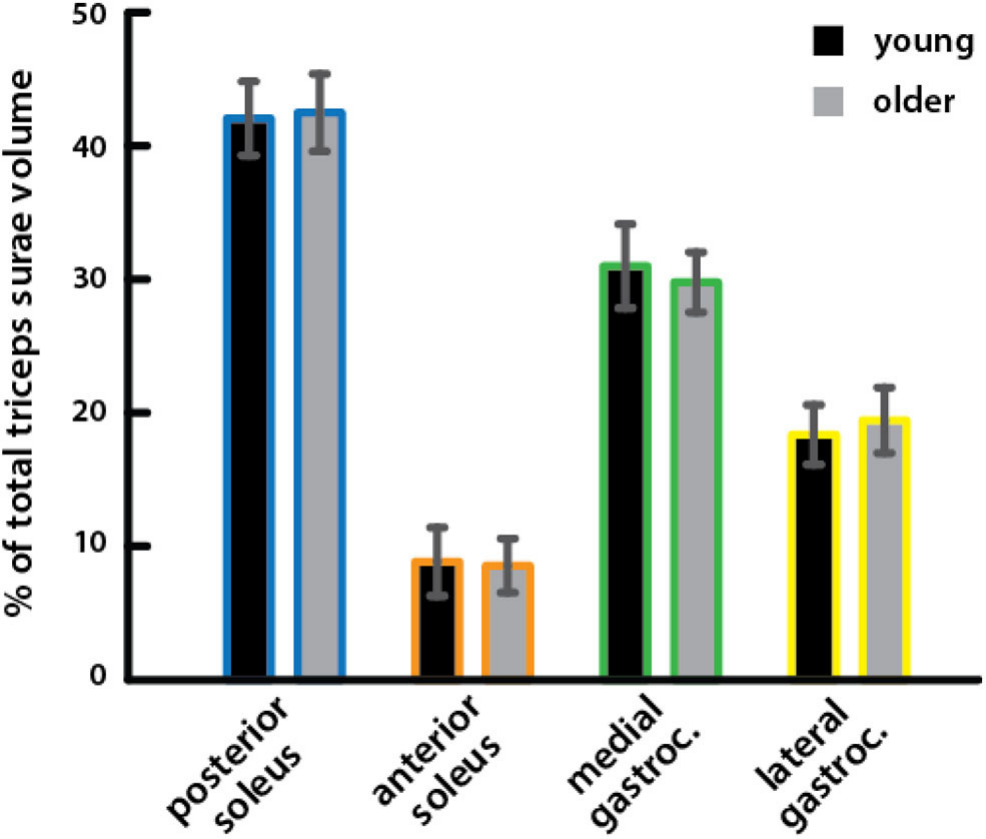
Relative volumes of triceps surae muscles did not differ between young and older adults. Relative volumes of individual triceps surae muscles as a percentage of the total triceps surae volume are shown for young (black) and older (gray) adults. A Mann–Whitney rank sum test detected no significant differences between young and older adults for any muscle (Table 2).

Relative volumes of individual triceps surae muscles, compared to total volume, were similar in young (percentage of total volume: *PS* = 41.9%, *AS* = 8.8%, *MG* = 30.9%, *LG* = 18.3%) and older (*PS* = 42.4%, *AS* = 8.5%, *MG* = 29.7%, *LG* = 19.4%) adults (Figure 2), with no significant difference for any muscle (Table 2).

**Table 2:** Triceps surae muscle volumes (mean ± standard deviation) compared between young and older adults.

		**Posterior soleus**	**Anterior soleus**	**Medial gastrocnemius**	**Lateral gastrocnemius**	**Total triceps surae**
Muscle volume (cm^3^)	Young	391.4 ± 84.3	83.8 ± 32.0	291.6 ± 81.9	175.1 ± 54.3	941.9 ± 229.9
	Older	329.4 ± 81.7	66.7 ± 20.5	231.1 ± 56.1	151.9 ± 47.2	779.1 ± 189.6
	*p*-value	0.668	0.668	0.576	0.436	0.167
Normalized volume (cm^3^/kg*m)	Young	2.93 ± 0.26	0.62 ± 0.19	2.17 ± 0.3	1.28 ± 0.21	7.00 ± 0.60
	Older	2.51 ± 0.35	0.52 ± 0.18	1.77 ± 0.3	1.15 ± 0.24	5.95 ± 0.93
	*p*-value	0.060	0.218	0.060	0.248	**0.015**
Relative volume (%)	Young	41.94 ± 2.76	8.83 ± 2.55	30.91 ± 3.13	18.32 ± 2.23	N/A
	Older	42.38 ± 2.89	8.53 ± 2.02	29.7 ± 2.25	19.39 ± 2.44	N/A
	*p*-value	1.588	1.588	1.256	1.256	N/A
Tendon CSA/volume (cm^−1^)	Young	1.58 ± 0.18e−3	8.38 ± 3.77e−3	2.15 ± 0.27e−4	3.71 ± 0.88e−4	6.62 ± 0.77e−5
	Older	2.09 ± 0.55e−3	1.14 ± 0.66 e−2	3.03 ± 0.10e−4	4.68 ± 1.56 e−3	8.91 ± 2.68e−4
	*p*-value	0.069	0.069	**0.048**	0.218	**0.019**

*Muscle volumes were normalized to account for body size differences by dividing by the product of each subject's height and mass. Relative volumes are the individual muscle volumes compared to the total triceps surae volume. Older adults had smaller absolute muscle volumes than young adults, but differences were not significant. The Mann–Whitney rank sum test was used to determine significance. The Holm–Bonferroni method was used to correct for family-wise error in tests repeated for individual muscle volumes. p < 0.05 was considered significant and appear in bold*.

### Triceps Surae Muscle Volumes Were Correlated With Body Size and Were Smaller per Body Size in Older Adults

**Figure 3:**
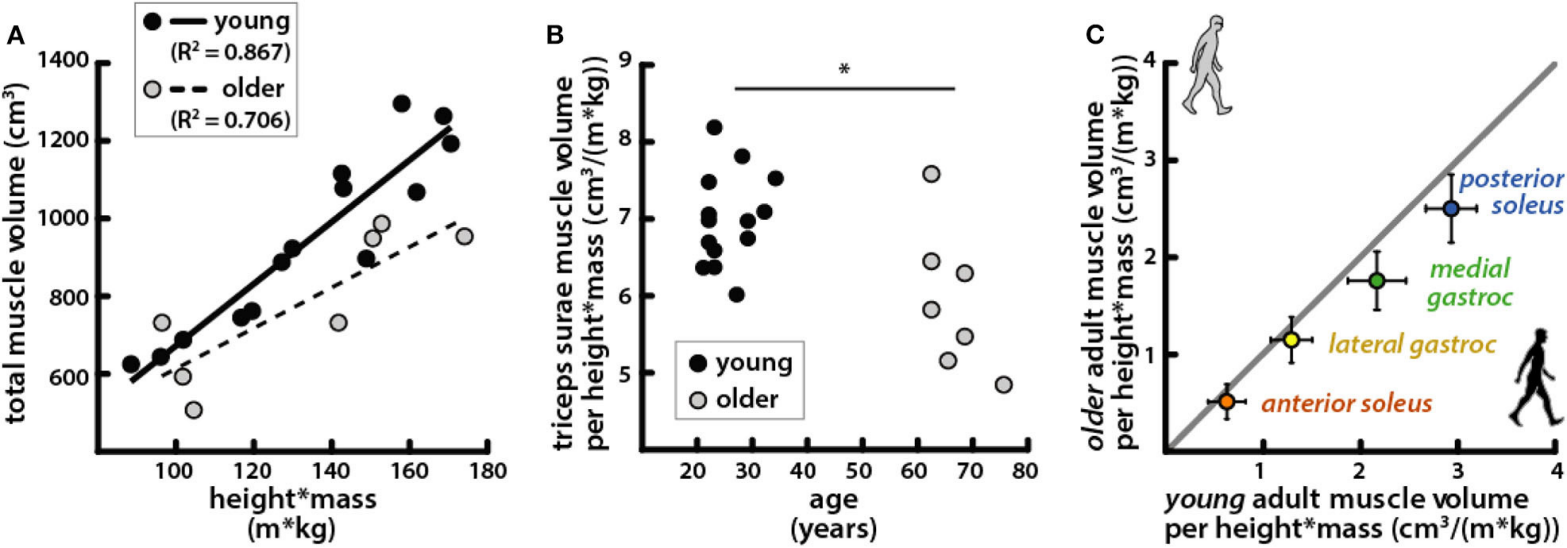
Triceps surae muscle volumes per body size were correlated with body size and were smaller per body size in older adults. **(A)** Total triceps surae volumes vs. the product of height and mass for young (black circles, solid line: *R*^2^ = 0.867, *p* = 1.33e−6) and older (gray circles, dashed line: *R*^2^ = 0.706, *p* = 0.018) adults. **(B)** Total triceps surae muscle volumes normalized by height * mass vs. age; a Mann–Whitney rank sum test determined muscle volume normalized by body size was significantly different between young and older adults (*p* = 0.015). **(C)** Average individual triceps surae muscle volumes normalized by height * mass for older adults vs. young adults; error bars indicate standard deviation. Points falling below the unity line (gray) indicate muscles that are smaller in older adults than in young adults (Table 2). *Indicates significant difference between young and old adults.

Total triceps surae volume was positively correlated with the product of height and mass in both young and older adults (Figure 3A). The scaling relationship between total triceps surae muscle volume and body size as well as the relationships for all individual muscles in young adults and older adults is provided in Table 3. Triceps surae volumes normalized by height * mass were smaller in older adults (Figure 3B), and all individual muscles were smaller per body size in older adults (percentage difference: *PS* = 15.4%, *AS* = 17.9%, *MG* = 20.3%, *LG* = 10.7%) (Figure 3C). Triceps surae muscle volumes were not significantly different between young and older adults but trended toward being smaller in older adults (percentage difference: *PS* = 17.2%, *AS* = 22.7%, *MG* = 23.1%, *LG* = 14.2%) (Table 2).

**Table 3:** Linear regression was used to determine the relationship between individual and total muscle volumes and body size, calculated as height * mass; Achilles tendon CSA; peak plantarflexion torque during walking at 1.25 m/s; and peak Achilles tendon force, estimated by dividing peak torque by moment arm, in young and older adults.

			**Posterior soleus muscle volume (cm^**3**^)**	**Anterior soleus muscle volume (cm^**3**^)**	**Medial gastrocnemius muscle volume (cm^**3**^)**	**Lateral gastrocnemius muscle volume (cm^**3**^)**	**Total triceps surae muscle volume (cm^**3**^)**
Height * mass (kg*m)	Young	*R*^2^	0.801	0.412	0.713	0.812	0.867
		Equation	0.29*x* + 22.09	0.54*x* + 88.64	0.28*x* + 53.05	0.45*x* + 55.7	0.11*x* + 31.26
		*p*-value	**<0.001**	**0.013**	**<0.001**	**<0.001**	**<0.001**
	Older	*R*^2^	0.762	0.253	0.658	0.465	0.706
		Equation	0.33*x* + 24.39	0.75*x* + 81.81	0.44*x* + 29.79	0.4*4*x + 64.81	0.14*x* + 26.36
		*p*-value	**0.010**	0.250	**0.027**	0.092	**0.018**
Achilles tendon CSA (mm^2^)	Young	*R*^2^	0.749	0.229	0.838	0.602	0.798
		Equation	0.13*x* + 12.17	0.18*x* + 46.01	0.14*x* + 21.47	0.17*x* + 30.73	0.05*x* + 16.56
		*p*-value	**<0.001**	0.084	**<0.001**	**0.001**	**<0.001**
	Older	*R*^2^	0.041	0.027	0.033	0.028	<0.001
		Equation	0.02*x* + 59.84	0.06*x* + 61.84	−0.02*x* + 70.75	−0.02*x* + 69.28	0.00*x* + 65.26
		*p*-value	0.662	0.726	0.697	0.721	0.983
Peak plantarflexion torque (Nm)	Young	*R*^2^	0.808	0.294	0.644	0.745	0.801
		Equation	0.26*x* + 14.03	0.41*x* + 80.12	0.24*x* + 45.65	0.38*x* + 47.39	0.09*x* + 26.22
		*p*-value	**<0.001**	**0.045**	**<0.001**	**<0.001**	**<0.001**
	Older	*R*^2^	0.725	0.279	0.650	0.707	0.760
		Equation	0.29*x* + 10.9	0.71*x* + 58.41	0.40*x* + 14.05	0.49*x* + 31.10	0.13*x* + 6.75
		*p*-value	**0.015**	0.223	**0.029**	**0.018**	**0.010**
Estimated peak tendon force (N)	Young	*R*^2^	0.765	0.163	0.621	0.668	0.723
		Equation	5.44*x* + 329.21	6.62*x* + 1,904.10	5.04*x* + 988.16	7.89*x* + 1,077.13	1.94*x* + 631.56
		*p*-value	**<0.001**	0.153	**<0.001**	**<0.001**	**<0.001**
	Older	*R*^2^	0.313	0.167	0.653	0.204	0.406
		Equation	4.19*x* + 1,078.59	12.19*x* + 1,645.40	8.81*x* + 423.04	5.85*x* + 1,569.86	2.05*x* + 857.57
		*p*-value	0.191	0.363	**0.028**	0.309	0.124

*The equation and R^2^-value for each linear regression are reported. p < 0.05 was considered significant and appear in bold*.

### Tendon CSA Was Correlated With Body and Muscle Size in Young Adults but Not in Older Adults

**Figure 4:**
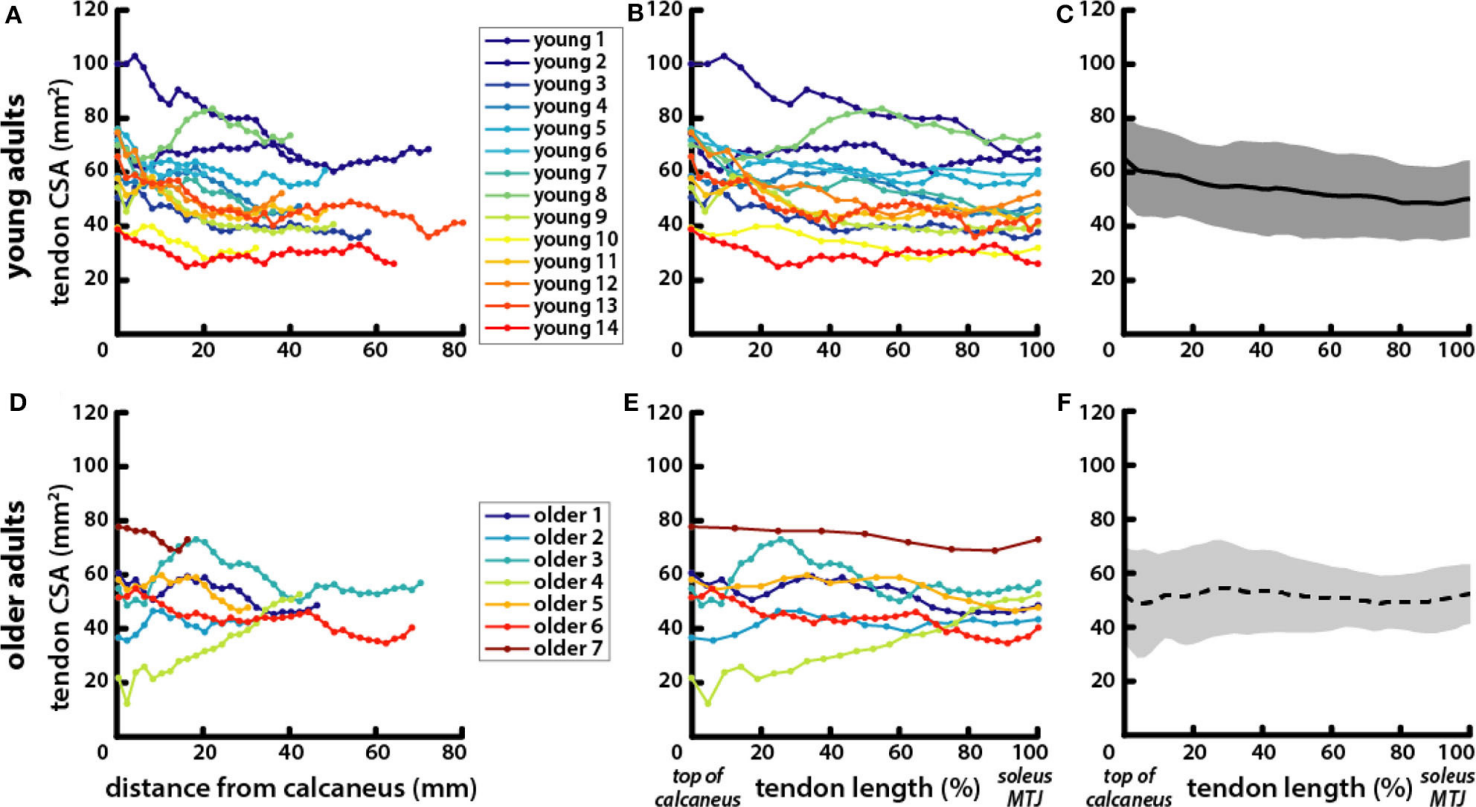
Tendons exhibited greater variation in length and CSA within age groups than between young and older adults. Achilles tendon CSA was measured in axial MR images of **(A)** young and **(D)** older adults from the most proximal image where the calcaneus was visible to the most distal image where the soleus was visible. CSA locations are reported as their distance from the calcaneus in the proximal–distal direction. To account for variations in free tendon length, CSA locations were normalized to length for **(B)** young and **(E)** older adults with the top of the calcaneus being the most distal point at 0% and the soleus MTJ the most proximal point at 100%. Average tendon CSA is plotted with normalized tendon length in **(C)** young (solid line, gray shading for standard deviation) and **(F)** older (dotted line, light gray shading) adults.

Achilles tendon CSA varied along its length from the top of the calcaneus to the soleus MTJ (Figure 4). There were no significant differences in average CSA between young (53.71 ± 14.88 mm^2^) and older (51.22 ± 13.18 mm^2^) adults or in CSA measured at the top of the calcaneus (young = 64.87 ± 16.34 mm^2^; older = 51.59 ± 18.02 mm^2^) and CSA measured at the soleus MTJ (young = 49.76 ± 14.05 mm^2^; older = 51.83 ± 10.89 mm^2^). Free tendon lengths also varied greatly between individuals but were not significantly different between young (50.95 ± 17.94 mm) and older (45.26 ± 20.39 mm) adults (Figures 4A,D). There were no significant differences in free tendon volume between young (2.65 ± 1.05 cm^3^) and older (2.25 ± 1.07 cm^3^) adults. CSA at half of the free tendon's length was used for further analysis and is subsequently referred to as Achilles tendon CSA.

**Figure 5:**
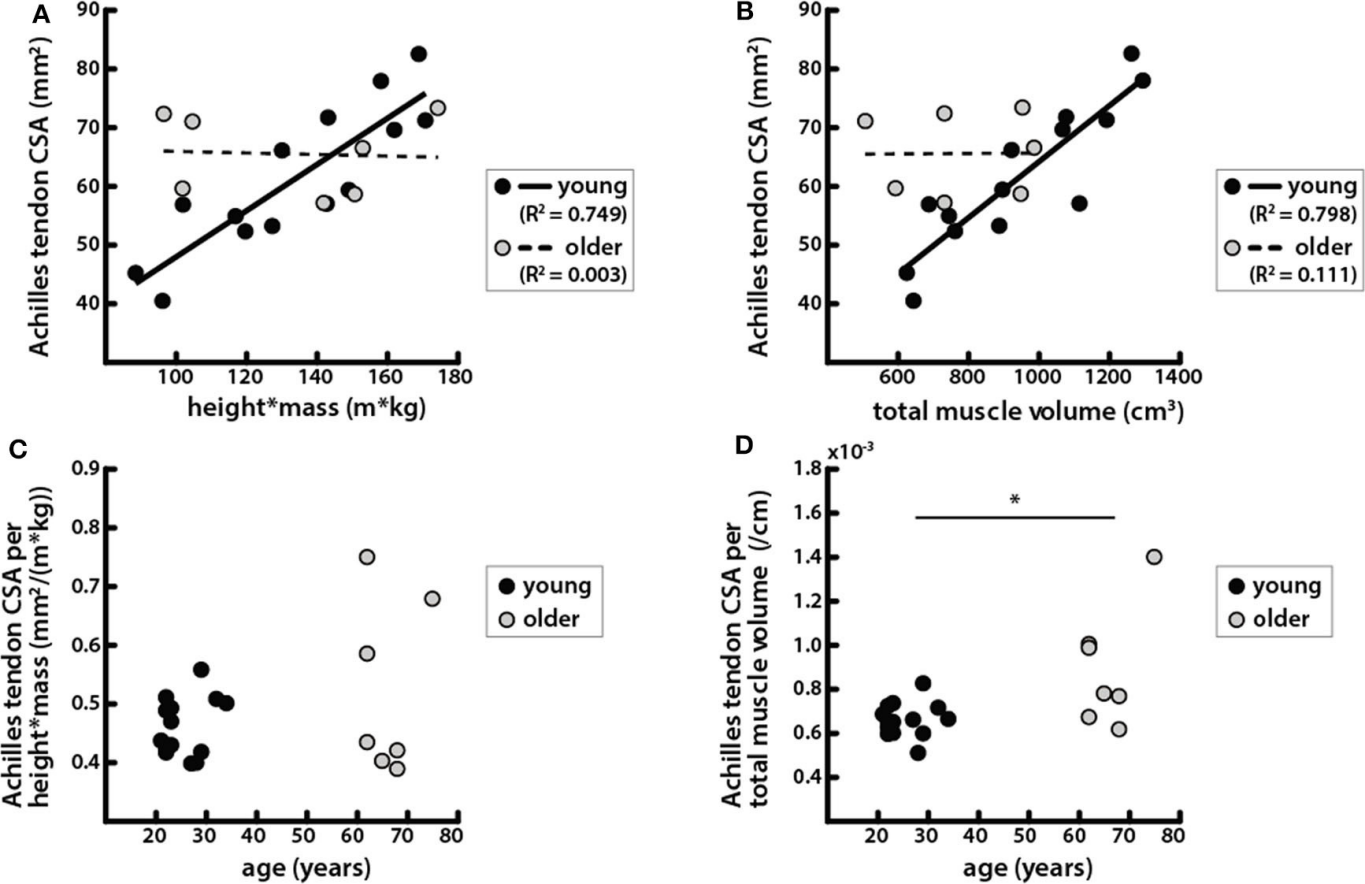
Achilles tendon CSA was correlated with body and muscle size in young adults but not in older adults. **(A)** Achilles tendon CSAs vs. the product of height and mass in young (black circles, solid line: *R*^2^ = 0.749, *p* = 6.42e−5) and older (gray circles, dashed line: *R*^2^ = 0.003, *p* = 0.901) adults. **(B)** Achilles tendon CSA vs. total triceps surae muscle volume in young (black circles, solid line: *R*^2^ = 0.798, *p* = 1.69e−5) and older (gray circles, dashed line: *R*^2^ = 0.111, *p* = 0.139) adults. **(C)** Achilles tendon CSA normalized by height * mass vs. age; a Mann-Whitney rank sum test determined that there were no significant differences between age groups in tendon CSA normalized by body size (*p* = 0.737). **(D)** Achilles tendon CSA normalized by total triceps surae muscle volume vs. age; a Mann–Whitey rank sum test determined that tendon CSA normalized by muscle size was significantly different between young and older adults (*p* = 0.019). *Indicates significant difference between young and old adults.

Achilles tendon CSA trended toward being larger in older adults, but the difference was not significant (Table 1). Tendon CSA was positively correlated with height * mass in young adults but not in older adults (Figure 5A). Similarly, tendon CSA was positively correlated with total triceps surae muscle volume in young adults (Figure 5B), while CSA was not related to total muscle volume in older adults.

Tendon CSA normalized to height * mass increased with age but was not significantly different between young and older adults (Figure 5C). However, tendon CSA normalized to total triceps surae muscle volume was significantly greater in older adults (Figure 5D). Additionally, the ratio of tendon size to muscle size was greater in older adults for all individual muscles, though the difference was only significant for the MG (Table 2).

### Peak Ankle Plantarflexion Torque During Walking Was Correlated With Body and Muscle Size in Young and Older Adults but Was Correlated With Tendon Size Only in Young Adults

**Figure 6:**
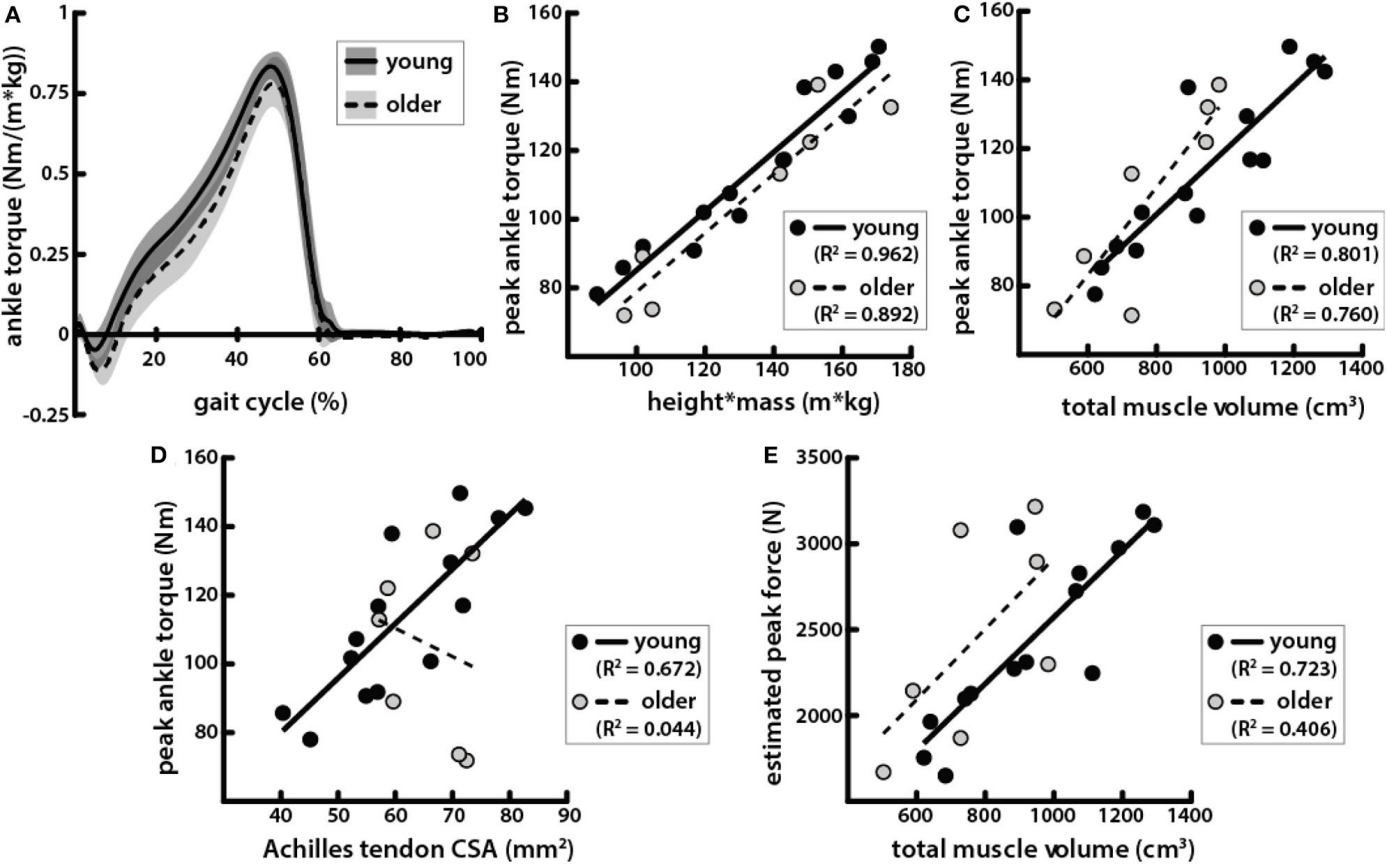
Peak ankle plantarflexion torque during walking was correlated with body and muscle size in young and older adults but correlated with tendon size only in young adults. **(A)** Average ankle torque normalized by body size, calculated as the product of height and mass, in young (solid line, gray shading for standard deviation) and older (dotted line, light gray shading) adults walking at 1.25 m/s plotted over one gait cycle. **(B)** Peak plantarflexion torque during walking at 1.25 m/s vs. the product of height and mass in young (black circles, solid line: *R*^2^ = 0.926, *p* = 3.77e−8) and older (gray circles, dashed line: *R*^2^ = 0.892, *p* = 1.35e−3) adults. **(C)** Peak torque vs. total triceps surae muscle volume in young (black circles, solid line: *R*^2^ = 0.801, *p* = 1.56e-5) and older (gray circles, dashed line: *R*^2^ = 0.760, *p* = 0.010) adults. **(D)** Peak torque vs. Achilles tendon CSA in young (black circles, solid line: *R*^2^ = 0.672, *p* = 3.31e−4) and older (gray circles, dashed line: *R*^2^ = 0.044, *p* = 0.651) adults. **(E)** Estimated peak forces, calculated by dividing peak torques by Achilles tendon moment arms, vs. total triceps surae muscle volume in young (black circles, solid line: *R*^2^ = 0.723, *p* = 1.16e−4) and older (gray circles, dashed line: *R*^2^ = 0.406, *p* = 0.124) adults.

Peak torque during walking at 1.25 m/s was positively correlated with height * mass (Figure 6B) and was not significantly different between age groups (Table 1, Figure 6A). Peak torque was positively correlated with total triceps surae muscle volume in young and older adults (Figure 6C). Peak torque was positively correlated with Achilles tendon CSA in young adults but not in older adults (Figure 6D).

### Estimated Peak Achilles Tendon Force Was Correlated With Triceps Surae Muscle Volume in Young Adults but Not in Older Adults

Achilles tendon moment arms were slightly smaller in older adults compared to young adults, but the difference was not significant (Table 1). Estimated peak Achilles tendon forces were also positively correlated with the total triceps surae volume in young adults (Figure 6E). However, in older adults, estimated peak force was not significantly correlated with total triceps surae muscle volume.

## Discussion

We investigated how triceps surae muscle volumes and Achilles tendon CSA correlate with each other, body size, and peak ankle plantarflexion torque during walking and determined how those relationships differ between age groups. Although peak plantarflexion torques did not differ between young and older adults walking at the same speed, we found evidence of sarcopenia as triceps surae muscle volume per body size was lower in older adults. However, the relative volumes of the triceps surae muscles were the same in young and older adults. Both young and older adults' triceps surae muscle volumes were positively correlated with both body size and peak walking torques, but triceps surae muscle volumes were positively correlated with Achilles tendon size only in young adults.

We found that volume distribution between the gastrocnemius and soleus was similar between age groups and furthermore that the volume distributions between the unique heads and compartments within the respective muscles were also similar between age groups (Figure 2). The distribution of triceps surae muscle volume between the soleus and the heads of the gastrocnemius is similar to what has been reported previously in young adults (Albracht et al., 2008). A previous study found that although physiological CSA (PCSA) distribution of the gastrocnemius heads and soleus within the triceps surae was similar in young and older adults, volume distribution between these muscles differed with age (Morse et al., 2005). The authors considered the soleus as a whole and did not image the full muscle, estimating volume of the distal portion using local multiple regression in each subject. Our findings were not consistent with our hypothesis that we would see age-related differences in volume distribution between the individual muscles of the triceps surae, which we expected due to the different functional roles of each muscle. Specifically, we expected that the gastrocnemius would experience greater atrophy because it is composed of a higher percentage of fast twitch fibers than the soleus (Johnson et al., 1973; Nilwik et al., 2013). Additionally, we posited that differences in gait between young and older adults might be explained by an altered balance of force production between muscles with different contributions to propulsion and support in walking (Neptune et al., 2001; Anderson and Pandy, 2003; McGowan et al., 2008; Francis et al., 2013). Older adults can respond to biofeedback to match the propulsive forces that young adults produce during walking but also increase support forces (Franz et al., 2014). If the soleus contributes more to support while the gastrocnemius contributes more to propulsion (Francis et al., 2013), a greater soleus-to-gastrocnemius size ratio could correspond with greater support to propulsive force generation. However, we did not see a redistribution of muscle volumes that would correspond with an imbalance between propulsive and support forces.

Older adults exhibited a proportional relationship between triceps surae muscle volumes and body size (Figure 3A) but with a lower ratio of muscle volume to body size than that in young adults (Figures 3B,C). This is consistent with prior work in healthy young adults which showed that lower-limb muscle volumes scale with body size, computed as the product of height and mass (Handsfield et al., 2014). In our current work, we found that the posterior and anterior compartments of the soleus each exhibit a scaling relationship between their volume and height * mass, which has been previously unexplored. Our results demonstrate that this relationship is generally preserved with sarcopenia but with a smaller scaling factor. In previous studies, individual triceps surae muscle volumes were found to also be predicted by the product of the maximum anatomical CSA and the muscle length when combined with a shape factor (Albracht et al., 2008; Karamanidis et al., 2019). Interestingly, a unique shape factor was required to accurately predict muscle volumes in older adults, as the use of the shape factor for muscle volumes measured in young adults (Albracht et al., 2008) overestimated volumes of older adult muscles (Karamanidis et al., 2019). Our work provides further evidence that while the muscle scaling relationships that are present in young adults are not eliminated by aging, they are certainly altered.

Achilles tendon CSA scaled with body size as well as with triceps surae muscle volumes in young adults (Figures 5A,B), which is consistent with the hypothesis that there is a mechanical relationship between muscle loading and tendon adaptation that determines CSA (Bohm et al., 2015). The correspondence of tendon CSA with muscle volumes that we have shown provides morphological evidence of the structure–function relationship between muscle and tendon in young adults. This work complements previous mechanical evidence showing correlations between normalized Achilles tendon stiffness and tendon forces estimated during maximum isometric voluntary plantarflexion contractions (Arampatzis et al., 2007). The lack of relationship between muscle and tendon size in older adults suggests that an alternative mechanism may be driving tendon adaptation. Altered tendon adaptation does not appear to result in a positive correlation between CSA and age; we did not find that Achilles tendon CSA was significantly larger in older adults (Figure 4C) as has been found in previous studies (Onambele et al., 2006; Stenroth et al., 2012). Those studies also found that tendon material properties change with age, showing that older adults have more compliant tendons. Increases in tendon CSA may be an adaptation to conserve the level of strain experienced in physiologic loading conditions in order to avoid failure, which occurs at similar strain levels even as material properties vary (LaCroix et al., 2013). This theory is supported by our finding that older adults had a significantly larger ratio of tendon CSA to muscle volume compared with the young adults (Figure 5D). The relationship between tendon and muscle size can be used to assess how much older adults deviate morphologically from young adults (Figure 5B) and may be indicative of the quality of the structure–function relationship between the Achilles tendon and triceps surae muscles in older adults. Such a metric might provide a more biomechanically relevant way to estimate the functional potential of adults, instead of the number of years they have been alive.

Peak plantarflexion torques during walking were positively correlated with body size, and the ratio of peak torque to height * mass was very similar in young and older adults (Figure 6B). This result is not surprising since the mechanical demands of walking are tied to body size. Body mass determines the load applied at the center of mass, while height is proportional to the lever arm of that load. Peak plantarflexion torques have been shown to differ between young and old adults when walking at the same speed (DeVita and Hortobagyi, 2000; Franz and Thelen, 2015; Boyer et al., 2017). Older adults in our studies did not walk with significantly lower peak torques, but it should be noted that the heights and masses of the young and older adults were similar in our study and also that they walked at a speed of 1.25 m/s, which was slower than in some previous studies (DeVita and Hortobagyi, 2000; Boyer et al., 2017). We did observe age-related differences in spatiotemporal parameters (Table 1). Peak torques during walking observed here were positively correlated with triceps surae muscle volumes (Figure 6C). Previous studies have shown positive correlations in young adults between plantarflexion muscle volumes and maximal plantarflexion torques measured both isokinetically and isometrically with a dynamometer (Morse et al., 2004; Baxter and Stephen, 2014), but the correlations were not as strong as those found in this study. In one of these studies, torque measured during maximum voluntary isometric contraction in older adults was not found to be significantly correlated with muscle volumes (Morse et al., 2004).

Achilles tendon CSA was positively correlated with body and muscle size, as well as with peak plantarflexion torque in young adults, but was correlated with none of these in older adults (Figure 6D). These results indicate that the mechanical relationships governing tendon size may differ between young and older adults. Additionally, estimated Achilles tendon forces were not correlated with triceps surae muscle volumes in older adults. We expected that accounting for moment arms would improve correlations between plantarflexor output and muscle size by removing variation due to differences in musculoskeletal geometry such that muscle size would be compared directly to force production. In a previous study, Achilles tendon moment arms were shown to positively correlate with plantarflexion torque measured by dynamometry, as well as with plantarflexor muscle volumes (Baxter and Stephen, 2014). In our study, moment arms were not directly correlated with muscle volumes, body size, or peak plantarflexion torques during walking in either young or older adults. Older adults in our study had slightly smaller moment arms than the young adults, as has been shown previously (Rasske and Franz, 2018), though the difference was not significant here.

There are several limitations of this study that should be mentioned. First, we were limited by a relatively small sample size, especially in the older adult group. Furthermore, the average age of the older adults in this study (66 ± 5 years) may have been too low to be associated with substantial age-related changes in function, considering adults can be considered in “midlife” under the age of 65 years old (Boyer et al., 2017). Previous authors have posited that age-related changes in tendon and muscle exhibit different time courses, as they observed lower muscle strength but similar tendon properties in older adults in their sixties (Karamanidis and Arampatzis, 2006). The observed age-related differences in muscle–tendon relationships may vary in older adults in their late seventies and eighties. Also, we did not control for physical activity level between the two age groups. All older adults reported engaging in regular physical activity, and while seven of the young adults also engaged in regular physical activity, we did not have reports from the other seven subjects, so we cannot confirm a similar activity level between groups. We may have been more likely to see differences in peak plantarflexion torque between young and older adults in a group over 75 years old. However, we did see differences between triceps surae muscles and Achilles tendons between our age groups, suggesting that structural changes may precede mobility deficits that arise with aging. Here, we used volume as a metric of muscle size, and therefore strength, since we did not measure fascicle or sarcomere lengths necessary for computing PCSA. Because the ratio of muscle belly length to muscle fiber length in the triceps surae was shown not to differ with age (Morse et al., 2005), we assumed volume remains reasonably proportional with strength in a comparison between young and older adults. Furthermore, moment arms used to estimate peak force were measured at an ankle position of 0° rather than the joint position at which peak torque occurred. However, peak torque occurred at very similar plantarflexion angles, so we do not believe this contributed to differences in estimated peak force between young and older adults.

This study reveals that the relationship between plantarflexor muscle and tendon structure may differ between young and older adults. The distribution of individual muscle volumes within the triceps surae was similar between young and older adults, as was the positive correlation between muscle volume and both body size and ankle plantarflexion torque. However, the same was not true for Achilles tendon CSA; CSA was only clearly related to body size and plantarflexion torque in young adults. Structure–function relationships that seem to exist between the Achilles tendon and the triceps surae muscles in young adults are no longer evident in all older adults. It appears that mechanisms affecting Achilles tendon morphology may be somehow altered with aging. Future work aimed at identifying factors affecting muscle–tendon relationships may provide a target for efforts to improve mobility in aging adults.

## Data Availability Statement

The datasets generated for this study are available on request to the corresponding author.

## Ethics Statement

The studies involving human participants were reviewed and approved by University of Wisconsin-Madison Health Sciences Institutional Review Board. The patients/participants provided their written informed consent to participate in this study.

## Author Contributions

KK, AE, JM, DT, and SB designed the study. AE and IL collected the data. KK analyzed the data, prepared the original draft, and created the figures. KK, AE, JM, DT, and SB interpreted the data. KK, AE, JM, IL, DT, and SB reviewed, edited, and contributed to the text. DT and SB secured the funding. All authors contributed to the article and approved the submitted version.

## Conflict of Interest

The authors declare that the research was conducted in the absence of any commercial or financial relationships that could be construed as a potential conflict of interest.
